# First person – Isabelle Toussaint-Lardé

**DOI:** 10.1242/bio.062141

**Published:** 2025-07-18

## Abstract

First Person is a series of interviews with the first authors of a selection of papers published in Biology Open, helping researchers promote themselves alongside their papers. Isabelle Toussaint-Lardé is first author on ‘ The impact of size and ontogeny on suction feeding kinematics in the axolotl (*Ambystoma mexicanum*)’, published in BiO. Isabelle is a PhD student in the lab of Anne-Claire Fabre at Universität Bern, Bern, Switzerland, investigating evolutionary biology and animal behaviour, especially herpetology.


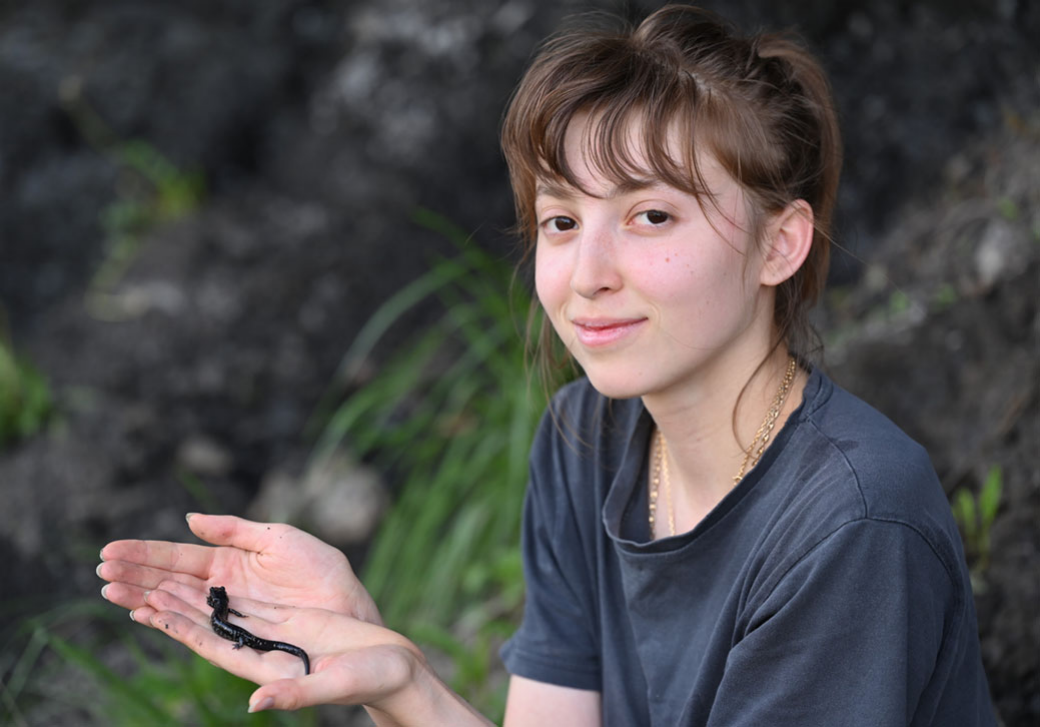


**Isabelle Toussaint-Lardé, during fieldwork in USA, holding a slimy salamander in her hand (image credit: A.-C. Fabre).**

**Describe your scientific journey and your current research focus**

In my current research, I investigate the interplay between feeding function and head morphology in salamanders and newts throughout ontogeny. My goal is to disentangle the effects of various factors, including experimental conditions, life stages, life cycle variations, and behavioural plasticity, on the feeding system and strategy.

At the beginning of my PhD, I focused on collecting high-speed videos of salamanders capturing their prey. This gave me the opportunity to do fieldwork in the USA, where there is a huge diversity of species. Now, I analyse these videos to extract kinematic variables that quantify head movements of salamanders while they are feeding. Meaning I watch a lot of salamanders and newts as they eat their meals! Depending on the research question, I perform various statistical analyses on these kinematic variables. I also read a lot of the existing literature to identify similar findings in other species and to support the interpretation of my results.

I watch a lot of salamanders and newts as they eat their meals!

**Who or what inspired you to become a scientist?**

During school, I was always fascinated by life and earth sciences. So, I naturally continued in this field during higher education. I developed a particular interest in herpetology and animal behaviour, which led me to an internship focused on studying salamanders. That experience was crucial, as it made me realize not only how much I enjoyed working directly with live animals, but also how much I appreciate the process of writing a scientific article, especially the discussion section, where I can connect my findings with those of other researchers.

**How would you explain the main finding of your paper?**

Axolotls are paedomorphic, which means that when they become adults, they retain features usually seen only in larvae (such as external gills). Moreover, at larval, juvenile or adult developmental stage, axolotls use the same method to catch their prey: suction feeding. This involves rapidly expanding the mouth cavity, which creates a flow of water that draws the prey into the mouth.

Because adult axolotls look and feed like larvae and juveniles, one might expect that the possible differences in how they capture prey would be due to their larger size.

To test this, we measured the feeding movements of the head such as the speeds and accelerations of the jaw and hyoid, or how wide the mouth opens and the hyoid depresses, and associated timings and durations.

We found that for most of these variables, values increased steadily with size, regardless of whether the individual was a larva, a juvenile, or an adult. However, for some variables related to speed and acceleration, the pattern was different: the increase was steady between larvae and juveniles, but for adults, the values increased more sharply with size. For these variables, size alone did not explain the differences we observed, but something happened at adulthood that modify the feeding movements.

These results suggest that adult axolotls are able to catch bigger and more elusive prey than larvae and juveniles. From an ecological perspective, this could mean that even though larvae and adults share the same environment (in the wild, they are endemic to the Xochimilco lakes), they may not compete for food, because they may be targeting different types of prey.

**What are the potential implications of this finding for your field of research?**

My study is part of a broader project investigating the role of life-cycle variation in the evolution of diversity, using salamanders as a model. Salamanders exhibit the greatest diversity of life-cycle types among tetrapods. Most species are biphasic, with an aquatic larval stage followed by metamorphosis into a terrestrial adult stage. However, some salamander species have evolved a ‘simplified’ life cycle, such as paedomorphic species, where adults retain larval traits and remain aquatic all their lives.

Metamorphosis is a widespread phenomenon in the animal kingdom and is thought to be advantageous because larvae and adults occupy different environments, reducing competition between larval and adult individuals within a same species. In life cycles such as paedomorphic, individuals avoid the costs of morphological and environmental changes, but larvae and adults share the same habitat and potentially compete for food.

Our results suggest that even in paedomorphic species there may be adaptations that reduce competition between life stages, contrary to expectations.

Furthermore, *Ambystoma mexicanum* is a highly endangered species with only a few individuals remaining in the wild. Thus, if the shift in diet across development is confirmed, understanding how axolotls feed through their ontogeny could be crucial for conservation efforts. Breeding programs could take this into account by varying the elusiveness of prey according to developmental stage, helping adult axolotls to express their natural behaviour before reintroduction into the wild, once their habitat is restored.

Our results suggest that even in paedomorphic species there may be adaptations that reduce competition between life stages

**Figure:**
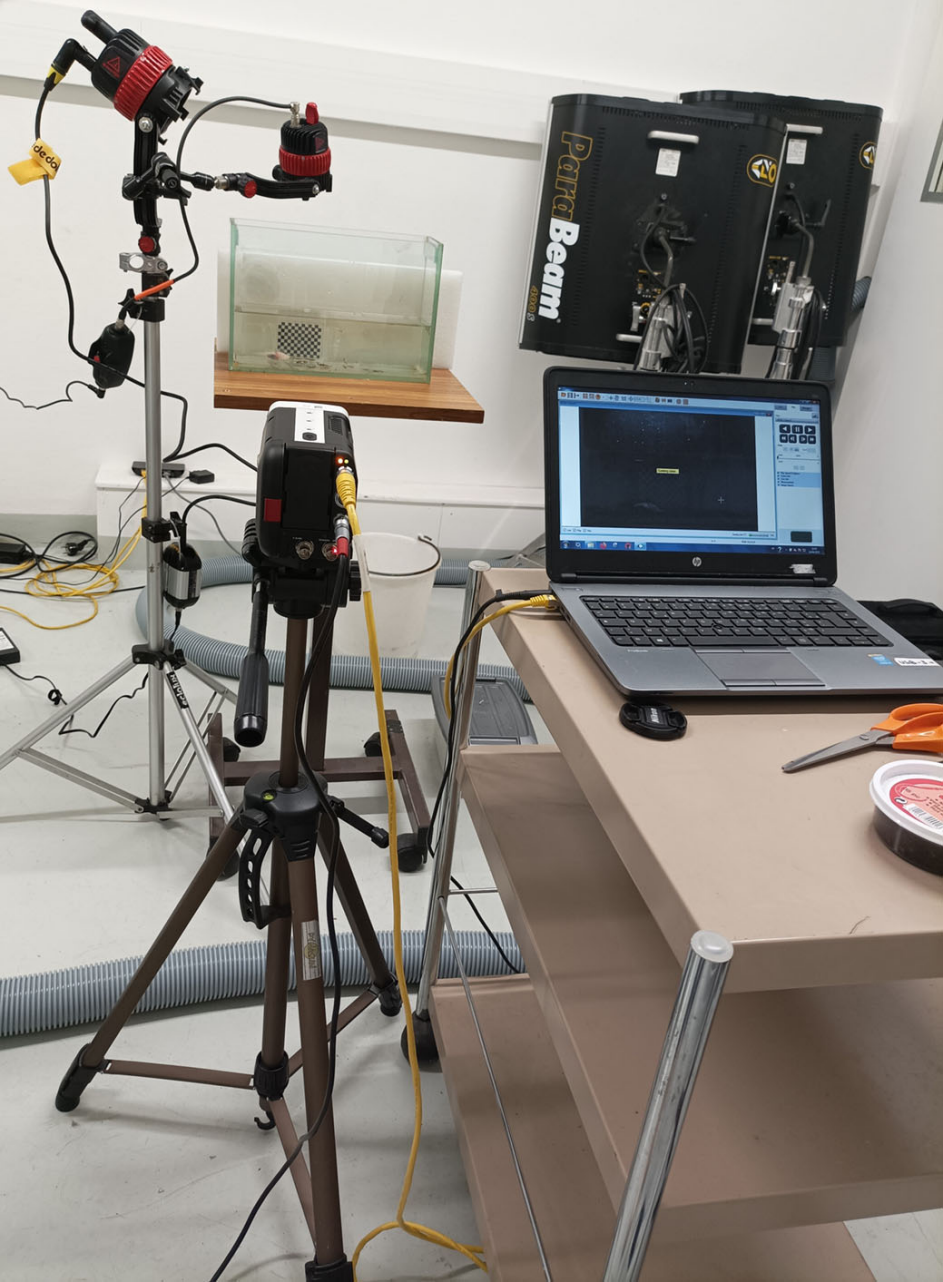
Setup used to film axolotls.

**Which part of this research project was the most rewarding?**

This research project was rewarding for several reasons. I felt particularly gratified to receive a grant to present the results at the World Congress of Herpetology in 2024. It was the first time I attended such a large conference and gave a talk, which made me very proud. However, I think the most rewarding part is that this is my first study as a PhD student and my first publication as a first author.

**What do you enjoy most about being an early career researcher?**

What I enjoy most about being an early career researcher is the opportunity to travel abroad for fieldwork and conferences, as I did not have the opportunity to travel that much before my PhD. I also appreciate being able to focus fully on my PhD research without the responsibilities of managing a team, as more senior researchers like PIs often have to do.

**What piece of advice would you give to the next generation of researchers?**

I would advise the next generation of researchers to make time for themselves and prioritize their mental health. In research, it's easy to feel overwhelmed by project deadlines, and if you are not careful, you can fall into the trap of working all the time, which can lead to burnout.

**What's next for you?**

I am still considering whether to pursue an academic career. I have two more years left in my PhD to explore my options. I am passionate about my topic and have planned many studies to understand how different factors influence salamander feeding kinematics, so for now, I hope to complete as many as possible. After my doctorate, I could then apply for a postdoc position to further develop the aspects that I was unable to fully explore in my doctoral thesis.
